# Townes-Brocks syndrome: genotype-phenotype correlations of *SALL1* variants in our series and the literature

**DOI:** 10.1038/s41431-025-01855-4

**Published:** 2025-05-10

**Authors:** Fiona Leduc, Perrine Brunelle, Fabienne Escande, Nassima Ramdane, Laurence Bellengier, Léa Giacomello, Christine Lefevre, Aurélie Mezel, Charlotte Samaille, Rony Sfeir, Philippine Toulemonde, Rare Disease networks, Catheline Vilain, Sebastian Neuens, Julie Soblet, Elise Schaefer, Olivia Boyer, Radka Stoeva, Alissandre Lecordier, Mathilde Nizon, Bertrand Isidor, Solène Conrad, Laëtitia Lambert, Mélanie Berard-Cloteau, Maria K Haanpää, Minna Toivonen, Sahar Mansour, Mohamed Wafik, Shereen Tadros, Abid Sharif, Lewis Darnell, Khaoula Zaafrane-Khachnaoui, Lucile Riera-Navarro, Fanny Morice-Picard, Klaus Dieterich, Alicia Coudert, Sophie Blesson, Anne-Marie Guerrot, Sacha Weber, Kara Ranguin, Sabine Sigaudy, Olga Glazunova, Geneviève Baujat, Sarah Grotto, Sébastien Moutton, Audrey Putoux, Hélène Vallin, Sylvie Manouvrier-Hanu, Catherine Vincent-Delorme, Florence Petit, Clémence Vanlerberghe

**Affiliations:** 1 CHU Lille, University Lille, Clinique de génétique « Guy Fontaine », ULR7364 RADEME, F-59000 Lille, France; 2 CHU Lille, University Lille, Institut de Génétique médicale, ULR7364 RADEME, F-59000 Lille, France; 3 CHU Lille, University Lille, Biochimie et Biologie moléculaire, ULR7364 RADEME, F-59000 Lille, France; 4 CHU Lille, Department of Biostatistics, F-59000 Lille, France; 5 SSR Pédiatrique Marc Sautelet, Médecine Physique et de Réadaptation Fonctionnelle, F-59650 Villeneuve d’Ascq, France; 6 CHU Lille, Endocrinologie pédiatrique, Centre de Référence DEV-GEN, F-59000 Lille, France; 7 CHU de Lille, Service de chirurgie orthopédique de l’enfant, F-59000 Lille, France; 8 CHU Lille, Department of Pediatric Nephrology, Hôpital Jeanne de Flandre, F-59000 Lille, France; 9 CHU Lille, University Lille, Division of Gastroenterology, Hepatology and Nutrition, Department of Pediatrics, Lille, France, F-59000 Lille, France; 10 CHU Lille, University Lille, Department of Otology and Neurotology, Lille, France; 11 Hôpital Universitaire Des Enfants Reine Fabiola, Hôpital Universitaire de Bruxelles, Université Libre de Bruxelles, Department of Genetics, Brussels, Belgium; 12 Hôpital Erasme, Hôpital Universitaire de Bruxelles, Université Libre de Bruxelles, Center for Human Genetics, Brussels, Belgium; 13 Université Libre de Bruxelles-Vrije Universiteit Brussel, Interuniversity Institute of Bioinformatics in Brussels (IB)2, Brussels, Belgium; 14 CHU Strasbourg, Institut de génétique médicale d’Alsace, Service de génétique médicale, F-67000 Strasbourg, France; 15 CHU Necker-Enfants Malades, University Paris Cité, Institut Imagine, CRMR MARHEA, F-75015 Paris, France; 16 Le Mans Hospital, Department of Medical Genetics, F-72000 Le Mans, France; 17 CHU Nantes, Service de Génétique Médicale, F-44000 Nantes, France; 18 CHRU Nancy, Service de génétique clinique, F-54000 Nancy, France; 19 Université de Lorraine, INSERM UMR_S1256, NGERE, F-54000 Nancy, France; 20 Turku University Hospital, Department of Genomics and Clinical Genetics, Turku, Finland; 21 St George’s University Hospitals, Clinical Genetics, London, UK; 22 Great Ormond Street Hospital for Sick Children, Clinical Genetics, London, UK; 23 Nottingham University Hospitals NHS Trust, Genomic and Molecular Medicine Service, Hucknall Road, Nottingham, NG5 1PB UK; 24 Centre Hospitalier Universitaire Nice, Université Côte D’Azur, Department of Medical Genetics, F-06000 Nice, France; 25 Pellegrin University Hospital of Bordeaux, Department of Paediatric Dermatology, F-33000 Bordeaux, France; 26 University Grenoble Alpes, Inserm, U1209, IAB, CHU Grenoble Alpes, F-38000 Grenoble, France; 27 CHU Grenoble, Génétique Clinique, F-38000 Grenoble, France; 28 CHU Tours, Service de Génétique, F-37044 Tours, France; 29 CHU Rouen, University Rouen Normandie, Department of Genetics and reference center for developmental disorders, Inserm U1245, F-76000 Rouen, France; 30 CHU de Caen, Service de Génétique et de Neurologie, F-14000 Caen, France; 31 AP-HM, Hôpital Timone Enfant, Département de Génétique Médicale, F-13000 Marseille, France; 32 Necker-Enfants Malades Hospital, Paris Cité University, Department of Genomic Medicine for Rare Diseases, French Reference Center for Constitutional Bone Diseases, INSERM UMR 1163, Imagine institute, F-75000 Paris, France; 33 AP-HP, Hôpital Trousseau, UF de génétique clinique, Centre de Référence anomalies du développement et syndromes malformatifs, F-75000 Paris, France; 34 Centre pluridisciplinaire de diagnostic prénatal (CPDPN) - MSPBordeaux Bagatelle, Maison des consultations, F-33400 Talence, France; 35 Hospices Civils de Lyon, Groupe Hospitalier Est, Service de Génétique, F-69500 Bron, France

**Keywords:** Disease genetics, Diseases

## Abstract

Townes-Brocks syndrome (TBS, MIM#107480) is an autosomal dominant disorder linked to *SALL1* alterations and characterized by a clinical triad (anorectal, thumb, and external-ear malformations), along with variable features. Renal failure and deafness can occur at any age, making follow-up essential. Some genotype-phenotype correlations have been suggested but data are limited. We collected clinical and molecular data from 49 patients with a *SALL1* (likely) pathogenic variant identified in our laboratory or through collaborations, and reviewed the 207 *SALL1* related-TBS patients previously reported in the literature. We performed statistical analysis to study genotype-phenotype correlations based notably on the variant position in relation to the glutamine-rich region. In our series, 25% of individuals presented with the clinical triad compared to 49.7% in the literature. The deafness frequency was similar (65%). Renal failure was diagnosed in 39.6% of our patients compared to 29.3% in the literature. Developmental delay or intellectual disability affected 9% of patients. Of the 22 *SALL1* variants in our series, 35% were located upstream of the glutamine-rich region, compared to 6.5% in the literature. Statistical analysis was performed on all patients, of which 26 and 200 carried a variant upstream and downstream of the glutamine-rich region, respectively. A significant increase in deafness, dysplastic ear, and thumb malformations and a significant decrease in renal failure were observed in the individuals carrying a variant located downstream of the region, but the patients were significantly younger. Future studies should aim to elucidate the complex pathophysiological mechanisms and prognosis of TBS, functionally and prospectively.

## Introduction

Townes-Brocks syndrome (TBS, MIM#107480) is an autosomal dominant disorder historically characterized by the triad of thumb, external ear and anorectal malformations [1]. Other features have subsequently been described, including deafness, feet malformations, kidney malformations and/or renal failure, congenital heart defects and genital anomalies in males [2–6]. Penetrance is reported to be complete but expressivity, including severity, is highly variable even within the same family [7, 8]. In particular, renal failure and deafness can occur at any age, making the follow-up of TBS patients essential [2, 9, 10].

TBS is due to heterozygous pathogenic variants of the *SALL1* (*Sal-like 1)* gene [11]. The SALL1 protein belongs to the Sal C2H2-type zinc finger protein family, characterized by zinc finger domains along the protein and a glutamine-rich region implicated in self-dimerization and heterodimerization with other Sall proteins [12–14]. SALL1 acts as a repressive transcription factor in developmental processes such as kidney, brain and limb development [13, 15]. Heterozygous *Sall1* knock-out mice have no phenotype, whereas ΔZn^2-10^ Sall1 mice, which produce a truncated protein, show some TBS features such as kidney malformations and hearing loss, suggesting a gain-of-function or dominant-negative effects of *SALL1* variants involved in TBS [16–18].

In human disease, most of the *SALL1* pathogenic variants involved in TBS are private and truncating [2, 3]. It has been demonstrated that some *SALL1* truncating variants were resistant to nonsense-mediated decay in TBS-patient fibroblasts and EBV-transformed B-cells, supporting the hypothesis that expression of a truncated protein is critical in the pathogenesis of TBS [16, 19]. However, few TBS patients carry a partial or complete deletion of *SALL1* with or without deletion of contiguous genes, likely resulting in haploinsufficiency. Genotype-phenotype correlations have been suggested but data are limited [2, 3, 20]. Among all the variants, a recurrent one, NM_002968.3:c.826 C > T p.(Arg276*), seems to be associated with a more severe phenotype with a higher prevalence of congenital heart defects [2, 21]. Furthermore, some authors support the association of haploinsufficiency, since some patients carry a *SALL1* deletion, with a milder phenotype [20]. It has been suggested that the variants located in the mutational hotspot towards the 5′-end are associated with a more severe phenotype than the variants located towards the 3′-end [2]. Furthermore, Wang et al. analyzed genotype-renal phenotype correlation according to zinc finger domains (*n* = 81) and concluded that variants affecting the *SALL1* gene positions from c.1500 to c.3468 are less likely to cause kidney phenotypes, although there is intrafamilial variability; however no statistical analyses were performed [22]. In a similar manner, genotype-phenotype correlations may be postulated depending on the domains remaining in the truncated-SALL1 protein. In particular, the presence of the glutamine-rich region, that plays a role in the dimerization of Sall proteins [12], has the potential to result in a dominant-negative effect, which may in turn manifest a more severe phenotype.

Here, we describe a new series of 49 patients from 22 families carrying a likely pathogenic or pathogenic heterozygous variant or a deletion of *SALL1* gene in order to better characterize TBS. We also review patients carrying a *SALL1* variant or deletion from the literature. We perform statistical analyses to establish possible genotype-phenotype correlations according to the position of the truncating variants, in relation to the glutamine-rich region and the mutational hotspot (towards 5′-end/3′-end).

## Subjects and methods

### Patients

Patients were recruited from the medical files of the laboratory at University Hospital of Lille (France) since 2006, and through calls for collaboration (AnDDI-Rares French network, ERN ITHACA and CRANIO European networks). Data were collected from a detailed form sent to the investigators and from the laboratory files when the analysis was performed locally. The inclusion criterion was the presence of a *SALL1* heterozygous truncating variant classified as likely pathogenic or pathogenic according to the ACMG classification [23], or a *SALL1* heterozygous deletion. Techniques for the genetic analyses were variable according to their evolution over the years and local diagnostic standards: Sanger sequencing, next generation sequencing, MLPA, chromosomal micro-array, exome sequencing (Table S1; precisions available on request). The variants in families 3, 4, 7, 9 and 17 have already been reported in a previous work from our team, but without detailed clinical description [24].

### Literature review

The literature review was performed using the HGMD Pro and Pubmed databases (last assessment October 2023) with the following terms: “Townes Brocks”;”*SALL1*”. As above, the inclusion criterion was the presence of a *SALL1* heterozygous truncating variant or deletion, classified as probably pathogenic or pathogenic. The exclusion criterion was the absence of a phenotype description.

### Genotype-phenotype correlations

Based on the literature data and previous genotype-phenotype studies in TBS, we tested two hypotheses according to the position of the truncating variant (start of the change in the case of a frameshift variant): 1) upstream or within (from c.1 to c.750) versus downstream (from c.751 to c.3975) of the glutamine-rich region (located between c.687 and c.750) [4], with the hypothesis of a dominant-negative effect leading to a more severe phenotype; 2) upstream versus downstream of the middle of the mutational hotspot (c.1164) [4], according to the hypothesis mentioned in the literature [2]. Patients carrying a *SALL1* deletion were excluded from this comparative analysis.

We also compared the phenotypes associated with *SALL1* deletion with those associated with truncating variants, excluding the patients with the recurrent p.(Arg276*) variant, previously associated with a more severe phenotype [21] to avoid introducing bias.

### Statistical analysis

Categorical variables are expressed as numbers (percentage). Continuous variables are expressed as means (standard deviation, SD) in the case of normal distribution or medians [interquartile range] otherwise. Normality of distribution was assessed using histograms and the Shapiro-Wilk test.

Comparative statistical analysis was performed on the data from this study and the literature. Comparisons between two groups relative to the variant location were performed by using Chi-square tests (or Fisher’s exact tests when expected cell frequency was <5) for categorical variables and Mann-Whitney U test for continuous variables. Variables compared were: sex, age, deafness, dysplastic ear, thumb malformation, malformation of lower limb, anorectal malformation, renal malformation, chronic renal failure, genital anomalies, congenital cardiac malformation and endocrinal anomalies.

Statistical testing was done at the two-tailed α level of 0.05. Data was analyzed using SAS software package, release 9.4 (SAS Institute, Cary, NC, USA).

## Results

### Present series

**Fig. 1:**
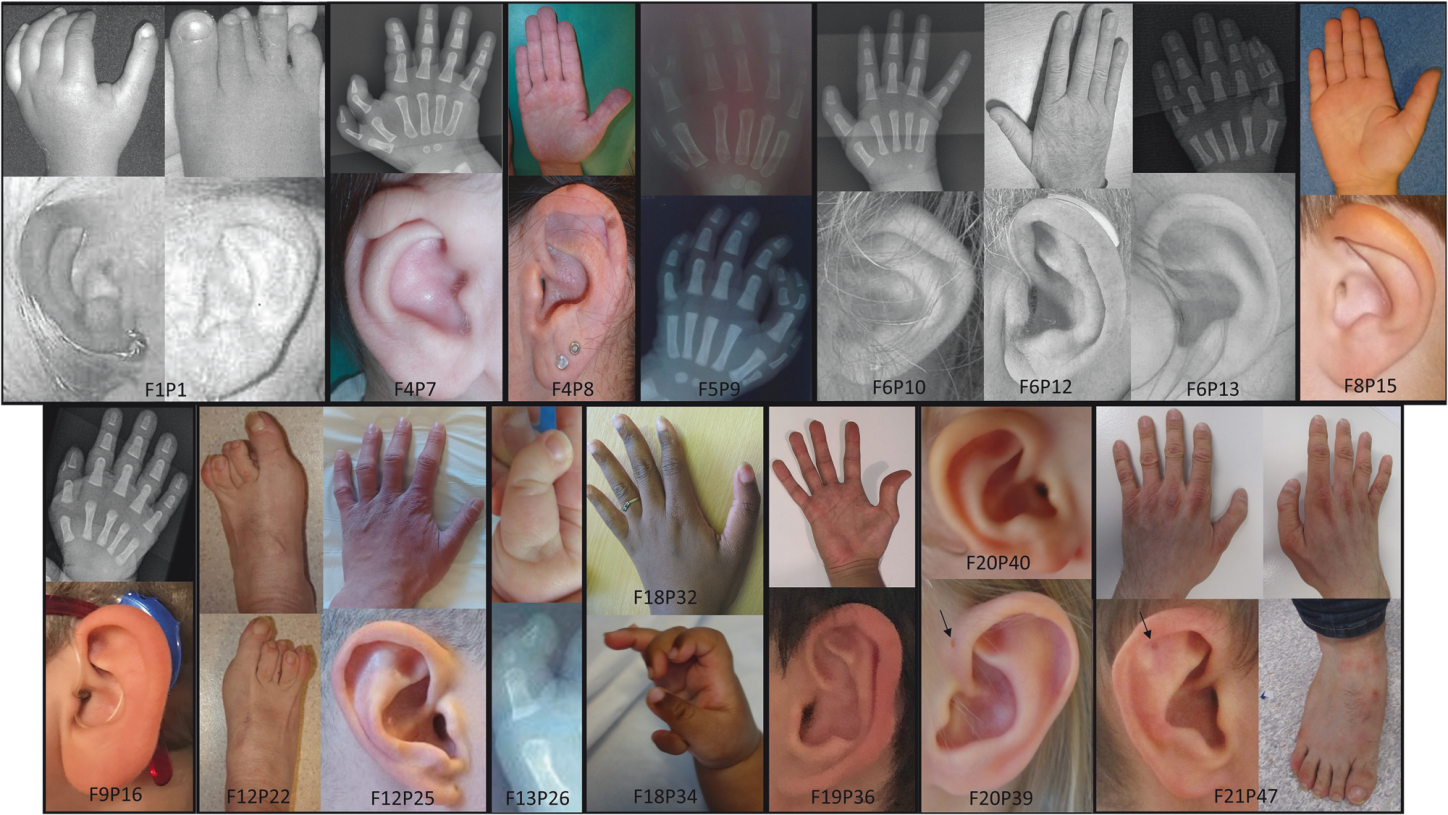
Ear dysplasia and limb malformations in 18 patients from 12 families in the present series. Ear dysplasia consisted mainly of abnormally folded helix and auricular tag or pit (arrow) (patient F20P40 was considered unaffected for ear dysplasia). Upper limb malformations mainly included triphalangeal thumb (patients F1P1, F5P9, F6P10, F6P13, F13P26, F18P32, F18P34, F19P36), duplicated thumb (patients F4P7, F5P9, F6P13, F9P16, F18P32, F18P34, F20O47), broad thumb (patients F4P8 and F20P47) (patients F6P12 and F8P15 were considered unaffected for upper limb malformations).

We recruited 49 TBS patients from 22 families, with a mean age of 23.6 ± 21.8 years old (Table 1, Table S1). The clinical triad (dysplastic ear, anorectal and thumb malformations) was observed in 25% of patients. The most common feature was dysplastic ear (69.4%), often a helix malformation (Fig. 1); thumb malformations were present in 53.1% with mostly triphalangeal and/or duplicated thumbs (Fig. 1); 62.5% of patients had an anorectal malformation. Lower limb malformations were mainly clinodactyly and overlapping toe (Fig. 1). Kidney structural malformations and chronic renal failure affected 53.8% and 39.6% of patients, respectively. Chronic renal failure occurred at any age, mostly in young adults but also in neonates (2 patients) and was often associated with hypo-dysplastic kidneys. Notably, 3 patients had chronic renal failure without abnormalities on kidney ultrasound. End-stage renal failure occurred in 6 patients, mostly adults. Deafness was diagnosed in 65.3% of patients. In most cases, deafness was post lingual, sensorineural, bilateral, mild to moderate. The course was sometimes progressive (4 patients). High frequencies were often affected, but audiograms results were heterogeneous. Six imaging studies of the ear were available: often normal, but incus and external auditory canal anomalies were seen in one patient each. Genital anomalies were observed only in males (6/25, 24%). Of note, 4 patients had hypothyroidism which was sometimes subclinical, and 5 had growth retardation. Regarding ophthalmologic features (16/44, 36.4%), 2/44 individuals had Stilling-Duane anomaly, 3/44 individuals acquired cataract, 8/44 individuals had refractive disorders; no coloboma or epibulbar dermoid were observed. Two patients from the same family (family 18) had tetralogy of Fallot, one of whom died at 3 months. Developmental delay (DD) and/or intellectual disability (ID) affected 6/40 (15%), often mild to moderate. Unfortunately, the intellectual quotient was rarely available (Table S2).

**Table 1:** Review of clinical and molecular data from the present series and the literature.

	Present study	Literature	TOTAL
**Clinical data**			
Sex ratio (F:M [missing data])	0.88 [0]	0.96 [9]	0.95 [9]
Age (m ± SD [missing data])	23.6 ± 21.8 [2]	15.6 ± 17.3 [114]	18.3 ± 19.2 [116]
Triad^a^ (%)	**12/48 (25.0)**	**95/191 (49.7)**	**107/239 (44.8)**
Dysplastic ear (%)	**34/49 (69.4)**	**162/194 (83.5)**	**196/243 (80.7)**
Thumb malformation (%)	**26/49 (53.1)**	**150/202 (74.2)**	**176/251 (70.1)**
Other malformation of upper limb (%)	8/49 (16.3)	22/201 (10.9)	30/250 (12.0)
Malformation of lower limb (%)	21/48 (43.8)	93/195 (47.7)	114/243 (46.9)
Ano-rectal malformation (%)	**30/48 (62.5)**	**130/195 (66.7)**	**160/243 (65.8)**
Deafness (%)	32/49 (65.3)	128/197 (65.0)	160/246 (65.0)
Kidney malformation (%)	21/39 (53.8)	81/191 (42.4)	102/230 (44.3)
Renal failure (%)	19/48 (39.6)	56/191 (29.3)	75/239 (31.4)
Genital anomalies (%)	7/48 (14.6)	27/198 (13.6)	34/246 (13.8)
Congenital heart defect (%)	8/35 (22.9)	37/196 (18.9)	45/231 (19.5)
Endocrine feature (%)	11/43 (25.6)	28/196 (14.3)	39/239 (16.3)
Ophthalmologic features (%)	16/44 (36.4)	29/194 (14.9)	45/238 (18.9)
Abnormal cranial nerve^b^ (%)	1/45 (2.2)	9/185 (4.9)	10/230 (4.3)
Facial asymmetry (%)	2/45 (4.4)	7/185 (3.8)	9/230 (3.9)
**Molecular data**			
*SALL1* variant located upstream or into the glutamine-rich region (%)	14/40 (35.0)	12/186 (6.5)	26/226 (11.5)
*SALL1* variant located upstream the middle of the mutation hotspot (%)	29/40 (72.5)	80/186 (43.0)	109/226 (48.2)
*SALL1* deletion (%)	9/49 (18.4)	19/205 (9.3)	28/254 (11.0)

Triad of thumb, external ear and ano-rectal malformations (in bold).

*F*, female; *M*, male; *m*, mean; *SD*, standard deviation.

^a^Triad of thumb, external ear and ano-rectal malformations. ^b^excluding Stilling-Duane anomaly.

**Fig. 2:**
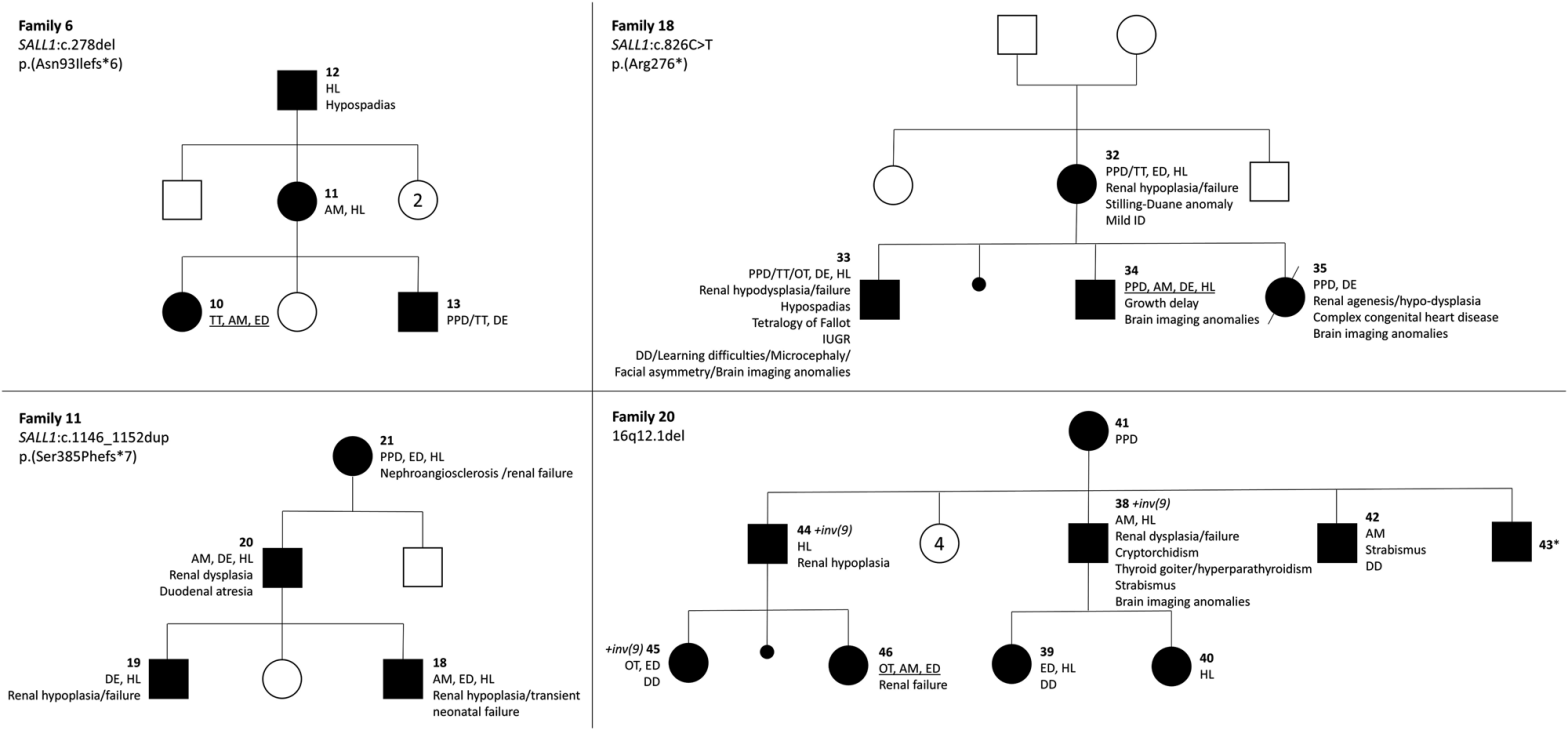
Pedigrees of 4 families in the present series. The first row refers to the four main signs: thumb features (preaxial polydactyly (PPD)/triphalangeal thumb (TT)/other thumb abnormality (OT)), anorectal malformation (AM), ear dysplasia (ED) and hearing loss (HL). The clinical triad is underlined when present. The numbers refer to the patient numbers given in Table S1, where the clinical and molecular characteristics are detailed. *No external abnormalities; cardiac and renal examinations were not performed. DD, developmental delay; del, deletion; dup, duplication; ID, intellectual disability; inv, inversion; IUGR, intra-uterine growth retardation.

Individuals with TBS show highly variable intrafamilial expressivity (Fig. 2). For example, there was no common clinical feature between all affected relatives in the three-generation family 6 (Fig. 2). In addition, the clinical spectrum may be mild, such as in patient F20P42 who had an anorectal malformation, non-specific moderate speech delay and strabismus, making the clinical diagnosis of TBS sometimes challenging. In the same family, patient F20P43 had no phenotypic features. However, the initial assessment was incomplete as it did not include renal and cardiac assessment.

**Fig. 3:**
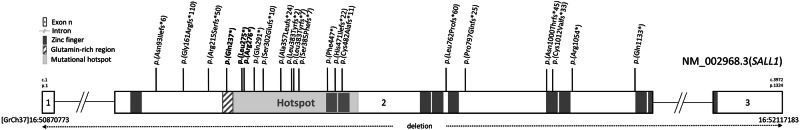
Schematic representation of the truncating variants and deletion of *SALL1* gene from the present series. All variants were located in exon 2. Fifty-two percent (11/21) of variants were located in the mutation hot-spot defined by Botzenhart et al. in 2007 [4]. Nineteen percent (4/21) of variants were located upstream or within the glutamine-rich region [4, 12], and 72.5% (29/40) of variants were located upstream of the middle of the mutation hotspot [4]. Three variants (bold) have been reported in other families in the literature [21, 25, 26]. The deletion involved *SALL1* and *HNRPNA1L3* genes.

Among the molecular data (Fig. 3), 9 members from one family carried a large 16q12.1 deletion (1.2 Mb) encompassing *SALL1* and *HNRPNA1L3* (absent from the OMIM database) protein-coding genes. Forty patients from 21 families carried frameshift or nonsense variants of which 18 had not been previously reported in the literature. Of note, one family (family 18) carried the recurrent variant c.826 C > T,p.(Arg276*) [21]. The variants c.709 C > T,p.(Gln237*) (family 12) and c.824 T > G;p.(Leu275*) (family 17) were reported once each [25, 26]. Among all, 14/40 (35%) patients carried a variant located upstream or within the glutamine-rich region, and 29/40 (72.5%) upstream of the middle of the mutational hotspot. No somatic mosaicism for a variant in the *SALL1* gene was identified in our series.

### Literature review

A total of 207 patients from 130 families (60 reports) were included in the literature review (Table S3). The mean age was 15.6 ± 17.3 years (missing data for 114 individuals). The clinical triad was identified in 95/191 (49.7%) of patients with dysplastic ears being the most common sign (83.5%), followed by thumb malformations (74.2%) and anorectal malformation (66.7%). Lower limb malformations were variable and affected 93/195 (47.7%) of patients. Kidney structural malformations and chronic renal failure affected 81/191 (42.4%) and 56/191 (29.3%) of patients (9.5% of which progressed to end-stage renal failure), respectively. Additionally, 128/197 (65.0%) of patients were diagnosed with deafness, which was often sensorineural and mild to moderate. A genital anomaly was observed in 24/96 (25%) of males and in only 3/93 (3.23%) of females. Endocrinological features were present in 28/196 (14.3%) of patients with 5.6% having hypothyroidism and 4% having growth retardation. Various ocular anomalies were observed: notably, 5 individuals had Stilling-Duane anomaly, 5 coloboma, 3 acquired or congenital cataract, and 3 epibulbar dermoid. Nearly 19% (37/196) of individuals had congenital heart defect. We undertook a review of patients with DD and/or ID and found a proportion of 8% (14/175) having DD/ID, after excluding patients with a double genetic diagnosis (one patient with mosaic trisomy 8), prematurity, neonatal resuscitation or only mild DD. For DD, when data was available, the severity is often mild; some patients have had good outcomes, particularly in the case of speech delay associated with deafness. The review of TBS patients with DD and/or ID from our series and the literature is available in Table S2.

Molecular data analysis revealed a deletion encompassing *SALL1* in 19/205 (9.3%), a truncating *SALL1* variant located upstream or within the glutamine-rich region in 12/186 (6.5%), a variant upstream of the middle of the mutational hotspot in 80/186 (43.0%) of patients.

### Genotype-phenotype correlations

Statistical analysis was performed on data from the present study (Table S1) and the literature (Table S3), comparing the clinical features of two groups defined by the localization of the truncating variant in relation to the glutamine-rich region: upstream and within (*n* = 26) or downstream (*n* = 200) of the region (Table 2). The prevalence of deafness (*p* = 0.037), dysplastic ear (*p* = 0.003) and thumb malformations (*p* = 0.003) was significantly higher when the variant was located downstream of the glutamine-rich region, whereas the prevalence of renal failure (*p* = 0.04) was significantly lower. Importantly, the patients were significantly younger and there were more missing data in the group “downstream of the glutamine-rich region”.

**Table 2:** Clinical comparisons of TBS patients carrying variants located upstream (*n* = 26) or downstream (*n* = 200) of the glutamine-rich region, and carrying variants located upstream (n = 109) or downstream (*n* = 117) of the middle of the mutational hotspot, from the present series and the literature.

Variable		Variant location					
		Upstream or into the glutamin-rich domain region	Downstream of the glutamin-rich domain region	*p* value	Upstream of the middle of the mutational hotspot	Downstream of the middle of the mutational hotspot	*p* value
Sex	Male (%)	13/25 (52.0)	99/193 (51.3)	0.95	61/105 (58.1)	51/113 (45.1)	0.056
	Female (%)	12/25 (48.0)	94/193 (48.7)		44/105 (41.9)	62/113 (54.9)	
Age	Available data	20/26	99/200	0.033*	70/109	49/117	0.36
	Median [Q1;Q3]	30.50 [4.000;44.00]	8.000 [2.670;28.00]		6.900 [2.250;35.00]	13.00 [5.000;31.00]	
Clinical features (%)	Deafness	11/24 (45.8)	130/193 (67.4)	0.037*	80/107 (74.8)	61/110 (55.5)	0.003*
	Dysplastic ear	14/24 (58.3)	162/190 (85.3)	0.003*	88/105 (83.8)	88/109 (80.7)	0.56
	Thumb malformation	13/26 (50.0)	146/196 (74.5)	0.009*	81/109 (74.3)	78/113 (69.0)	0.38
	Malformation of lower limb	11/25 (44.0)	94/189 (49.7)	0.59	62/107 (57.9)	43/107 (40.2)	0.009*
	Ano-rectal malformation	15/24 (62.5)	127/191 (66.5)	0.70	71/106 (67.0)	71/109 (65.1)	0.78
	Kidney malformation	6/17 (35.3)	88/188 (46.8)	0.36	48/99 (48.5)	46/106 (43.4)	0.46
	Chronic renal failure	12/24 (50.0)	55/188 (29.3)	0.040*	39/105 (37.1)	28/107 (26.2)	0.086
	Genital anomalies	2/26 (7.7)	30/191 (15.7)	0.38	23/108 (21.3)	9/109 (8.3)	0.007*
	Congenital heart defect	1/18 (5.6)	39/188 (20.7)	0.21	24/99 (24.2)	16/107 (15.0)	0.092
	Endocrine feature	7/23 (30.4)	29/190 (15.3)	0.079	18/106 (17.0)	18/107 (16.8)	0.975

Bold values indicate significant results.

*p < 0,05.

Q1;Q3, first and third interquartile; SD, standard deviation.

Additionally, we conducted a statistical analysis based on the mutational hotspot, comparing variants located upstream (*n* = 109) and downstream (*n* = 117) of the middle of the mutational hotspot (Table 2). We found a significant increase in deafness (*p* = 0.003), lower limb malformations (*p* = 0.009) and genital anomalies (*p* = 0,007) (sex ratio with *p* = 0.056) in individuals carrying a variant located upstream of c.1164.

Death occurred at 3 months in a patient (F18P35) with a severe phenotype associating complex congenital heart defect (pulmonary atresia, ventricular septal defect, major aortopulmonary collateral arteries, tetralogy of Fallot), unilateral kidney agenesis and multiple cystic changes in both anterior horns of the lateral ventricles with mild ventriculomegaly. She carried the recurrent variant c.826 C > T,p.(Arg276*) inherited from her mother. The phenotype associated with this variant is known to be more severe due to an increased frequency of congenital heart defects [21], estimated here at 46.2% (12/26), whereas only 15.6% (28/180) of individuals carrying other genotype (excluding *SALL1* deletions) were affected. Notably, this is the only variant associated with tetralogy of Fallot in 3 patients reported in the literature, one of whom also died [21, 27].

A total of 22 individuals were found to carry a *SALL1* deletion without any other OMIM-morbid gene(s) involved (including 9 patients from 1 family in our series), while 6 individuals carried a larger deletion. When the deletion only involved *SALL1* as OMIM-morbid gene, the proportions of all variables appeared to be decreased compared to individuals who carried a *SALL1* variant (excluding the recurrent variant c.826 C > T,p.(Arg276*) associated to a more severe phenotype), except for DD/ID which affected 6/20 (30%) patients.

## Discussion

We present a novel series of TBS involving 49 patients from 22 families, and compared their phenotypes to the previously reported patients. Whereas we observed similar complete penetrance and highly variable expressivity [2, 4–6, 8, 11] in both groups, the clinical triad was found in only 25% of our series compared with 49.7% of patients from the literature. Yan et al. [28] demonstrated that patients with typical-TBS are more prevalent in the second generation than in the first, possibly due to a mosaic state in the first generation. However, we do not support this observation because when evidence of de novo occurrence of a *SALL1* variant was provided (parental genetic analysis performed), the index case presented the characteristic clinical triad in our series. Furthermore, the pedigrees of the four families shown in Figure 2 suggest a highly variable intra-familial expressivity, rather than a generational effect possibly linked to somatic mosaicism in the first affected generation.

Besides, renal failure was more common in our series, perhaps due to an older age compared to the literature data and/or a better follow-up due to a better knowledge of the TBS course. Chronic renal failure is observed at any age, mostly in young adults but also in neonates. Whereas hypo-dysplastic kidneys are often associated, we noted, in 3 patients of our series, the occurrence of chronic renal failure without obvious kidney malformation, suggesting that a renal follow-up throughout the life is essential for TBS patients. *SALL1* variants have also been reported in patients with isolated kidney disease, highlighting the wide variability in the expression of *SALL1*-associated phenotypes [29].

Deafness associated with TBS appears to be mostly post lingual, sensorineural, bilateral, mild to moderate. The course was sometimes progressive. CT scan are more often normal but, as we noted incus and external auditory canal anomalies in 2 patients, Yan et al. recently reported malformations in the middle ear and enlargement of the vestibular system in one patient [28].

Cognitive impairment was reported in 20/215 (9%) of documented TBS patients. Unfortunately, the outcomes for most patients affected by DD and the intellectual quotient were rarely available. As *Sall1* has been implicated in neurogenesis in mice [30], this modest increase in DD/ID risk suggests that other factors are involved in the cognitive phenotype of TBS patients. Fifty-seven percent of patients in our series have prenatal ultrasounds findings, with intrauterine growth retardation, renal, limb and cardiac malformations. With the improvement of ultrasound scans and the increased use of prenatal genetic analyses [31], family counselling in relation to the prognosis becomes central. In this respect, it is essential to precise the outcome of TBS patients, which is still poorly known. Further prospective studies are therefore needed, in particular to clarify the neurodevelopmental trajectory of TBS patients, such as large-scale genetic analysis (exome or genome sequencing), since a significant proportion of patients may have a double genetic diagnosis [32, 33].

Regarding genotype-phenotype correlations, we hypothesized that the ability of the truncated protein to dimerize through the conservation of the glutamine-rich region may cause a more severe phenotype. This could be due to the escape from the nonsense-mediated mRNA decay process and a dominant negative effect. Here, we found a significantly higher prevalence of deafness, dysplastic ear and thumb malformations when the *SALL1* variant is located downstream of the glutamine-rich region in patients who were significantly younger (more missing data on age in this group), whereas deafness occurs with age. Kidney malformations seem to be more frequent also but without reaching statistical significance. Since the glutamine-rich region seems to play a role in the dimerization of Sal proteins, the above features could be partly due to a dominant negative effect, as suggested by other authors [12, 17]. Furthermore, Kiefer et al. showed that the truncated Sall1 protein is sufficient to cause preaxial polydactyly and triphalangeal thumb in transgenic mice (expression under the control of a limb-specific promoter), with this phenotype being more likely to manifest when a wild-type Sall1 is co-expressed [16]. Besides, the prevalence of renal failure is significantly lower when the variant is located downstream of the glutamine-rich region. Importantly, patients were significantly younger and there were more missing data on age in this group, which could lead to a confounding bias as renal failure occurs with age. Nevertheless, some data support the hypothesis that a stable truncated Sall1 protein, which is able to dimerize, may also have a partial function in kidneys later in life. *Sall1* is required during development [15, 34], but its expression is also reactivated after kidney injury [35]. Interestingly, Hirsch et al. observed that a mouse model expressing a truncated mutant of Sall1, containing the glutamine-rich region, is less susceptible to acute kidney injury than wild-type and knock-out *Sall1* mice [36]. This phenomenon could explain the better renal function in TBS patients with a *SALL1* variant located downstream of the glutamine-rich domain, expressing a more stable truncated Sall1 protein possibly capable of dimerization. We also performed a statistical analysis between two groups based on the localization of the variant in the mutational hotspot (upstream or downstream of the middle of the mutational hotspot, c.1164). As reported in the previous statistical analysis, we found a significant increase in the frequency of deafness in individuals carrying a variant located upstream of c.1164, suggesting that the pathophysiology of deafness may be related to nucleotide positions from c.751 to c.1164. We also confirmed that the recurrent p.(Arg276*) variant is associated with a more frequent congenital heart defect, including tetralogy of Fallot [21, 27]. In our series, this specific malformation had only been observed with this recurrent variant (family 18). Besides, Innoceta et al. mention a correlation between haploinsufficiency (*i.e*. intragenic deletion or large deletion involving only the *SALL1* gene, *n* = 10) and a milder phenotype, except for DD/ID. They compared patients carrying deletion to GeneReviews data [2], and patients carrying the recurrent p.(Arg276*) variant (*n* = 20). In this previous paper, a significant difference was seen only for dysplastic ears and congenital heart defects when compared to patients carrying the p.(Arg276*) variant, possibly due to lack of statistical power [20]. Thus, these previous and current genotype-phenotype correlations do not appear to be robust enough to be used in patient follow-up, especially as there is considerable intra-family variability (Fig. 2), but remain interesting for the study of TBS pathophysiology. Taken together these results suggest that *SALL1* variants could cause TBS either by affecting the ability of SALL1 to interact with SALL proteins and DNA through a resistance to nonsense-mediated decay and a dominant negative effect (truncating variants located downstream of the glutamine-rich domain), or through a dosage effect (deletion or truncating variants located upstream of the glutamine-rich domain).

To date, there is no international consensus on the management of TBS. With regard to the data collected in the literature review, our French multidisciplinary working group proposes an initial evaluation and follow-up of TBS patients regardless of the *SALL1* variant identified [37] (Table 3). Of note, in addition to the annual audiological and renal function monitoring previously recommended by J. Kohlhase [2], we advise annual monitoring of thyroid function, the usefulness of which will need to be reassessed.

**Table 3:** Proposal for initial assessment and follow-up in patients with TBS.

Concern	Initial assessment	Follow-up
Constitutional	• Psychomotor development, measurement of growth parameters	• Classical follow-up: psychomotor development, measurement of growth parameters, etc
Genetics	• Personal and familial past medical history • Clinical examination: features suggestive of TBS and differential diagnoses • Genetic analysis	• Genetic counselling according to patient and physician expectations • No systematic follow-up
Orthopaedic	• Clinical examination: thumb malformation and clubfeet, mobility and joint stability • Hand X-rays	• According to initial assessment • No systematic follow-up
Hearing	• Audiological evaluation • *+/− Ear CT-scan and/or MRI if deafness*	• According to initial assessment • **Systematic**: annual audiological evaluation
Gastro-intestinal	• Clinical examination: anorectal malformation	• According to initial assessment • No systematic follow-up
Renal	• Clinical examination: urinary functional signs, measurement of growth parameters and blood pressure • Blood tests: creatininemia, estimation of glomerular filtration rate *+/− complementary cystanine C* • Urine tests: proteinuria, creatinuria • Renal ultrasound • *+/− Cystography if suspected uropathy* • *+/− Scintigraphy or uro-MRI*	• According to initial assessment • **Systematic**: - Annual: measurement of growth parameters and blood pressure, blood tests (creatininemia, estimation of glomerular filtration rate), urine tests (proteinuria, microalbuminuria, creatinuria) - Every two years: renal ultrasound
Cardiac	• Clinical examination: signs of cardiac insufficiency, heart auscultation • Heart ultrasound • *+/− ECG, pulmonary X-rays*	• According to initial assessment • No systematic follow-up
Endocrine	• Clinical examination: measurement of growth parameters, external genitalia • Blood tests: TSH • *+/− Neonatal and kinetics during mini-puberty hormonal assessments, SRY, karyotype if bilateral cryptorchidism or posterior hypospadias*	• According to initial assessment • **Systematic**: - Clinical examination: measurement of growth parameters, hypothyroidism symptoms - Blood tests: TSH annually
Eyes	• Ophthalmologic assessment: visual behavior/acuity, refraction examination under cycloplegia, slit lamp and fundus examination, study of oculomotor skills	• According to initial assessment • No systematic follow-up

From Protocole National de Diagnostic et de Soins-Syndrome de Townes-Brocks, Haute Autorité de Santé [38].

CT-scan, Computed Tomography; MRI, Magnetic Resonance Imaging; TBS, Townes-Brocks syndrome.

The proportion of patients with typical TBS but no molecular diagnosis has not been studied here as there are no consensus criteria in the literature for assessing clinical diagnosis. The literature reports a proportion of 75% of molecular diagnoses in typical TBS patients (classic triad) [2]. This gap may be explained by differential diagnoses such as Duane-radial ray syndrome (MIM #607323), branchio-oto-renal syndrome (MIM #113650) or VACTERL association (MIM #192350). Besides, DACT1 variants have been associated with a phenotype known as Townes-Brocks syndrome 2 (MIM #617466), which causes mostly congenital anomalies of the kidney and urinary tract, possibly associated with skeletal, anorectal and genital anomalies [38, 39]. In addition, alteration of *SALL1* regulatory elements may be involved in molecular negative cases. In support of this hypothesis, C.A. Stevens et al. [40] found a deletion upstream of *SALL1* in a propositus with anorectal malformation, congenital anomalies of the kidney and urinary tract, broad thumbs and hearing loss compatible with TBS. Genome sequencing and functional studies should allow to elucidate non-coding molecular alterations and increase the diagnostic yield in TBS.

In conclusion, we found a genotype-phenotype correlation according to the variant location relative to the glutamine-rich region, involved in the dimerization of Sal proteins, for features that may appear at any age, such as deafness, and, with the bias of a significant difference in age and missing data, renal failure. In the future, functional and prospective studies are needed to elucidate the pathophysiological mechanisms and the outcomes of TBS in order to better monitor patients and treat them as early as possible to improve prognosis.

## Supplementary information

Supplementary Table S1: Clinical and molecular characteristics of 49 TBS patients from the present series.

Supplementary Table S2: Review of TBS patients with DD and/or ID from our series and the literature.

Supplementary Table S3: Clinical and molecular characteristics of 207 TBS patients from the literature.

## Data Availability

Molecular data have been submitted to the ClinVar database (accessions from SCV005880107 to SCV005880127). The datasets generated and analyzed in the current study are available from the corresponding author upon reasonable request.
